# Efflux at the Blood-Brain Barrier Reduces the Cerebral Exposure to Ochratoxin A, Ochratoxin α, Citrinin and Dihydrocitrinone

**DOI:** 10.3390/toxins13050327

**Published:** 2021-04-30

**Authors:** Matthias Behrens, Sabine Hüwel, Hans-Joachim Galla, Hans-Ulrich Humpf

**Affiliations:** 1 Institute of Food Chemistry, Westfälische Wilhelms-Universität Münster, 48149 Münster, Germany; mattbehrens@wwu.de; 2 Institute of Biochemistry, Westfälische Wilhelms-Universität Münster, 48149 Münster, Germany; huwel@wwu.de (S.H.); gallah@uni-muenster.de (H.-J.G.)

**Keywords:** blood-brain barrier, mycotoxins, ochratoxin A, ochratoxin α, citrinin, dihydrocitrinone, probenecid, efflux transporter, HPLC-MS/MS, porcine brain capillary endothelial cells

## Abstract

Recent studies have implied that environmental toxins, such as mycotoxins, are risk factors for neurodegenerative diseases. To act directly as neurotoxins, mycotoxins need to penetrate or affect the integrity of the blood-brain barrier, which protects the mammalian brain from potentially harmful substances. As common food and feed contaminants of fungal origin, the interest in the potential neurotoxicity of ochratoxin A, citrinin and their metabolites has recently increased. Primary porcine brain capillary endothelial cells were used to investigate cytotoxic or barrier-weakening effects of ochratoxin A, ochratoxin α, citrinin and dihydrocitrinone. The transfer and transport properties of the mycotoxins across the barrier formed by porcine brain capillary endothelial cell monolayers were analysed using HPLC-MS/MS. High levels of Ochratoxin A caused cytotoxic and barrier-weakening effects, whereas ochratoxin α, citrinin and dihydrocitrinone showed no adverse effects up to 10 µM. Likely due to efflux transporter proteins, the transfer to the brain compartment was much slower than expected from their high lipophilicity. Due to their slow transfer across the blood-brain barrier, cerebral exposure of ochratoxin A, ochratoxin α, citrinin and dihydrocitrinone is low and neurotoxicity is likely to play a subordinate role in their toxicity at common physiological concentrations.

## 1. Introduction

### 1.1. The Blood-Brain Barrier

The blood-brain barrier (BBB) represents a permeability barrier between the blood stream and cerebral endothelial cells. The BBB regulates the exchange of compounds between the blood circulation and the brain of most vertebrates. Furthermore, it protects the neurons from changes in osmotic pressure and xenobiotics or potentially harmful endogenous compounds in the bloodstream. Three types of cells form the BBB, which can also be referred to as the neurovascular unit: The cerebral microvessels are formed by endothelial cells, which surround the blood capillary. Their major function is the fundamental basis of the barrier controlling the influx of nutrients and the efflux of potentially harmful compounds [1]. This layer of endothelial cells is covered by a basal lamina, which also encloses pericytes. Astrocyte endfeet are located on this basal lamina and almost completely surround it. Pericytes and astrocytes exhibit various regulatory functions and control the tightness of the endothelial barrier as well as protein expression [2,3]. Furthermore, astrocytes support the cerebral microstructure and synthesise cholesterol for the electrical insulation of neuronal axons at the myelin sheath [4,5].

To maintain the major barrier functions, the barrier functionality can be divided into three barrier properties. Firstly, the so-called physical barrier is formed by tight junctions (TJ), which close the gaps between the endothelial cells and limit the paracellular diffusion to a minimum. TJ are formed by transmembrane proteins such as occludin or claudins, which are connected to the actin cytoskeleton. This physical barrier represents the actual barrier and is responsible for the high transendothelial electrical resistances (TEER) above 1000 Ω·cm² [4]. However, small lipophilic molecules can circumvent the physical barrier by transcellular diffusion through the cellular bilayer membranes. Various types of transport proteins form the second barrier, the transport barrier. The transport proteins protect neuronal tissues by transporting xenobiotics from endothelial cells back to the blood stream facilitating their excretion [4]. A third option to reduce cerebral exposure to xenobiotics is the metabolic barrier. Xenobiotics diffusing into the endothelial cells can undergo phase I and phase II metabolism by various enzymes. Especially the formation of highly polar conjugates such as sulfates or glucuronides reduces their ability to cross cellular barriers and facilitates faster excretion [1,4].

To mimic the BBB, primary porcine brain capillary endothelial cells (PBCEC) were introduced as a powerful and flexible model. They enable studies regarding the interactions of endothelial cells with pericytes [6] and astrocytes [7] and have been successfully applied for transfer studies of xenobiotics yielding results with good comparability to the human BBB in vivo [8,9]. Using PBCEC high TEER values above 1000 Ω·cm^2^ can be reached representing an excellent correlation with the BBB in vivo. By using hydrocortisone in physiological concentrations, the tightness of the barrier is enhanced by cytoskeletal rearrangements leading to improved TEER values [10,11].

The use of primary cells in the PBCEC model has the advantage that more morphological features of the brain endothelium are maintained compared to cell lines [3], however the protocol to prepare primary cells is rather laborious and time consuming. When comparing PBCEC, to the in vivo microenvironment at the BBB, the latter includes astrocytes, pericytes, microglia, neurons, and the blood flow which improves differentiation and polarization of the endothelial cells [12].

In previous publications using PBCEC ergot alkaloids produced by *Claviceps* easily permeated the BBB in vitro [13] and severe cytotoxic and barrier-impairing effects of the mycotoxins T-2 toxin and HT-2 produced by *Fusarium* species toxin were found. Furthermore, T-2 toxin and HT-2 toxin were shown to cross the BBB in vitro [14]. The type B trichothecenes deoxynivalenol and 3-acetyldeoxynivalenol as well as moniliformin crossed the BBB in this model. Deoxynivalenol also impaired the barrier integrity of the PBCEC monolayer [15].

### 1.2. Ochratoxin A and Ochratoxin α

**Figure 1:**
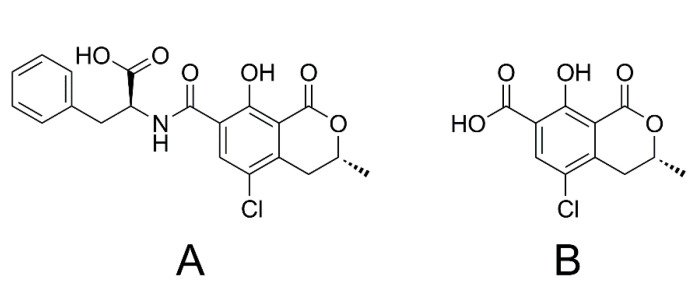
Chemical structures of OTA (**A**) and OTα (**B**).

Ochratoxin A (OTA, Figure 1A) is a common fungal contaminant produced by several fungal species of the genera *Penicillium* and *Aspergillus* [16]. Since these fungi colonise various food and feed before and after harvest of their raw products, OTA is found in numerous products [16,17]. Several genera of microorganisms (protozoa, bacteria, yeasts and fungi) as well as plant cells cleave the OTA amide bond, releasing ochratoxin α (OTα, Figure 1B) and L-phenylalanine. Therefore, OTα can be considered as a metabolic degradation product of OTA [17].

OTA is characterised by a diverse and highly species dependent metabolism. In contrast to other animal species, humans are considered to have a relatively low metabolic capacity for OTA. The most important metabolic pathways include the hydrolysis of the amide and the lactone moieties, hydroxylation and conjugation reactions. Therefore, OTα is a product of animal metabolism as well. Except for the open lactone OTA, the products of these reactions are considered to be less toxic than OTA [18].

A pilot study analysing the human OTA and OTα levels detected both compounds in urine and blood plasma samples of 13 German volunteers. Whereas no OTA conjugates were detected, OTα is supposed to be frequently metabolised and conjugated with sulfate and glucuronic acid moieties. The mean OTA concentration in blood plasma was 0.25 ± 0.03 ng/mL, whereas mean total OTα level in blood plasma was 2.99 ± 2.24 ng/mL [19]. Another recent study used the dried blood spot technique to analyse the whole blood levels of OTA in 50 German volunteers and found 0.21 ± 0.066 ng/mL as mean concentration [20].

OTA is well known for its nephrotoxic properties, which were tested in various animal species except adult ruminants [21]. Furthermore, OTA as well as aristolochic acid are associated with human Balkan endemic nephropathy (BEN), but neither can be directly linked to BEN [22].

In an animal study, upon feeding Wistar rats for up to 6 weeks with 289 µg OTA/kg body weight/48 h, OTA was detected in all areas of the brain and enriched in a time-dependent manner in concentrations up to 94 ± 16 ng/g in brain tissue [23]. Astrocytes and neurons from the ventral mesencephalon and cerebellum of rat brains were used to investigate OTA toxicity. IC_50_ values regarding protein and DNA synthesis ranged from 14 ± 2 µM to 69 ± 9 µM. OTA toxicity was higher in ventral mesencephalon compared to cerebellum and higher in neurons compared to astrocytes [24]. An acute intraperitoneal dose of 3.5 mg/kg body weight (10% of the LD_50_) to mice caused a depletion of striatal dopamine, increased oxidative stress and inhibited oxidative DNA repair [25]. Studies in neurons revealed the apoptotic potential of OTA in these cells and associated OTA with neurodegenerative diseases such as Parkinson’s and Alzheimer’s disease [26,27]. Furthermore, it has been shown that OTA affect the viability and function of astrocytes [28,29].

### 1.3. Citrinin and Dihydrocitrinone

**Figure 2:**
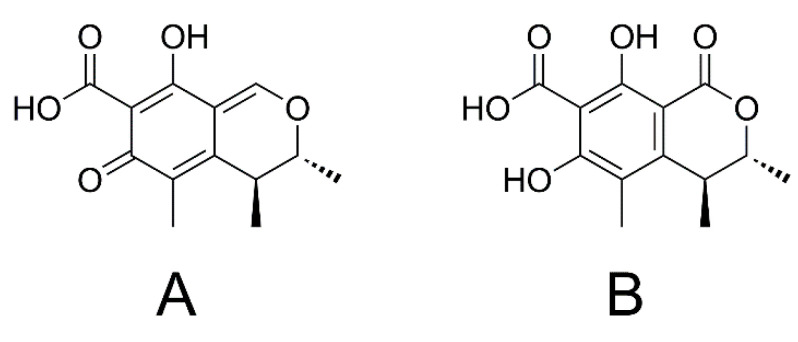
Chemical structures of CIT (**A**) and DHCIT (**B**).

Citrinin (CIT, Figure 2A) is a secondary metabolite of *Penicillium*, *Aspergillus* and *Monascus* grown on several cereals and a common co-contaminant of OTA [16,30]. It is often detected in grains, fruits, nuts and spices [16].

Besides thermal degradation products, the most important CIT derivative is dihydrocitrinone (DHCIT, Figure 2B). DHCIT is a conversion product of CIT in some *Penicillium* stains and might therefore be present in food and feed commodities [31]. Moreover, DHCIT is the most frequently detected mammalian CIT metabolite [32], which was also detected in human blood and urine samples from Germany [33,34,35], Bangladesh and Haiti [36,37].

As for OTA, CIT is known to be nephrotoxic and supposed to be involved BEN, but its mode of action remains unclear. Kidney is the most important toxicokinetic target of CIT, but it has also been shown to affect liver, erythrocytes and bone marrow [38,39,40]. Using probenecid (4-(*N*,*N*-dipropylaminosulfonyl)benzoic acid) as an organic anion transporter (OAT) inhibitor, CIT, but not DHCIT, was shown to be responsible for nephrotoxic effects after CIT uptake [41]. CIT induces the formation of reactive oxygen species (ROS) in vitro, which is discussed as a possible mode of action in CIT toxicity [42]. Recently CIT, DHCIT and OTA were compared regarding their toxic effects on V79 cells, revealing DHCIT to be less toxic than CIT. Therefore, the formation of DHCIT is considered as a detoxification reaction of CIT [43]. When focusing on their combined toxicity, OTA and CIT are known to act mostly additive or synergistic in various species and models [44]. Nevertheless, the question of whether CIT leads to increased oxidative stress in mutagen or genotoxic in vivo still remains inconclusive, because different studies yielded contradictory results [38].

### 1.4. Aim of the Study

This study focused on OTA and CIT, which are frequently detected co-occurring mycotoxins in food and feed, and their metabolites OTα and DHCIT. In this study it was evaluated whether OTA, OTα, CIT and DHCIT cause adverse effects on the BBB in vitro. In addition, it was studied, if and how fast the compounds can cross the BBB, to receive data about the potential exposure of the brain to OTA, CIT and their most common metabolites. Finally, the influence of the OAT inhibitor probenecid on the active transport of OTA, OTα, CIT and DHCIT was investigated in order to identify the characteristics of this transport. These data are needed to assess potential neurotoxic effects of the investigated mycotoxins.

## 2. Results

### 2.1. Viability Test

**Figure 3:**
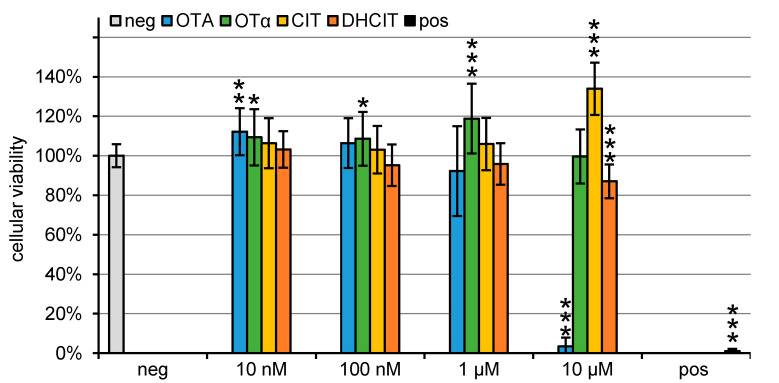
Cellular viability (n = 18, CCK 8-assay) of PBCEC after treatment with OTA, OTα, CIT and DHCIT for 48 h. Significant differences compared to the negative control (0.1% ACN) are marked (highly significant differences (*p* < 0.001) are marked with ***, differences with medium significance (*p* < 0.01) with ** and with low significance (*p* < 0.05) with *. T-2 toxin (10 µM) was incubated as a positive control.

To test the cytotoxicity of the investigated mycotoxins PBCEC were incubated with concentrations ranging from 10 nM to 10 µM for 48 h. The cellular viability was analysed using the CCK-8 assay (Figure 3). Representative microscopic images of PBCEC 48 h after mycotoxin application can be found in the Supplementary Materials (Figure S1).

T-2 toxin as a positive control showed a strong effect and the cellular viability was significantly (*p* < 0.001) reduced to 2 ± 1% compared to the negative control (grey column), which maintained a viability of 100 ± 6%, as described before [14].

Similar results were obtained for 10 µM OTA (blue bars), resulting in a significant (*p* < 0.001) reduction of the cellular viability to 3 ± 4% and were accompanied with very similar morphological changes of the PBCEC as they were observed for 10 µM T-2 toxin. OTA concentrations of 1 µM reduced the cellular viability to 92 ± 23. Only minor changes in cellular morphology were observed. Most cells maintained their characteristic shapes whereas a few cells showed the same abnormalities as the positive control and 10 µM OTA. As for all other mycotoxins tested in this study, no morphological changes were observed for 100 nM OTA and lower concentrations. Moreover, 10 nM OTA caused a significant increase (*p* < 0.01) of the dehydrogenase activity to 112 ± 12%. This weak effect should not be misinterpreted as an increase in cellular viability, since small numbers of primary cells tend to stronger variability resulting in higher standard deviation.

The incubation of 10 µM OTα (green bars) for 48 h resulted in a cellular viability of 100 ± 14%, which did not differ significantly compared to the negative control. Lower concentrations of 1 µM OTα revealed a significant (*p* < 0.001) increase to 119 ± 18%, whereas an increase after 10 nM and 100 nM OTα incubation reached low statistical relevance (*p* < 0.05).

Furthermore, 10 µM CIT (yellow bars) led to a significant (*p* < 0.001) increase in the dehydrogenase activity to 134 ± 13%. Reducing the CIT concentration to 1 µM resulted in 106 ± 13% dehydrogenase activity indicating no significant effect on the cellular viability.

After 48 h incubation with 10 µM DHCIT (orange bars), the cellular viability was significantly (*p* < 0.001) decreased to 87 ± 9%, whereas no significant differences were observed after incubation with 1 µM DHCIT resulting in 96 ± 11% compared to the negative control.

### 2.2. Barrier Integrity

**Figure 4:**
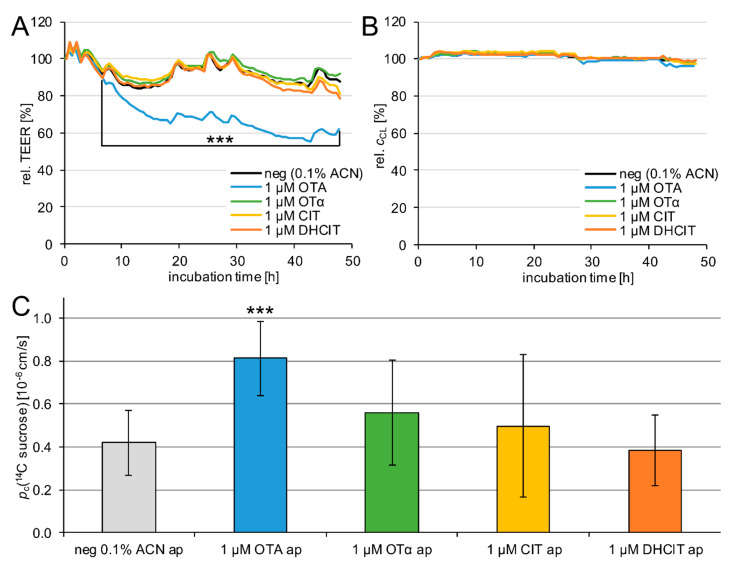
Relative TEER values (**A**, *n* = 9) and electrical capacitance *c*_CL_ (**B**, *n* = 9) of PBCEC after apical incubation of 1 µM toxin for 48 h and sucrose permeability *p*_c_ (^14^C sucrose) after 48 h (**C**, *n* = 18). Data are referred to the TEER and *c*_CL_ at t = 0 h. For diagram clarity, error bars are not shown in A and B. For TEER values the average standard deviations was 6% (max. 16%) and for *c*_CL_ 2% (max. 6%). The observed weak effects after 1, 2.5, 6.5, 18, 24, 28, 42, and 48 h in A and B are resulting from sampling for the transfer studies (highly significant differences (*p* < 0.001) are marked with ***.

In order to study the barrier integrity PBCEC were incubated with 1 µM OTA, OTα, CIT and DHCIT in the apical compartment (blood side) of Transwell^®^ filter inserts for 48 h. The TEER values as well as the electrical capacitance (*c*_CL_) were monitored by cellular impedance spectroscopy (Figure 4A,B). As a negative control the permeability of ^14^C sucrose was measured after 48 h. Sucrose is not transported by any cellular transport protein and represents a marker for an intact BBB. The resulting permeability coefficients are given in Figure 4C. 

As illustrated in Figure 4A, only OTA (blue line) significantly reduces the TEER (*p* < 0.001) continuously from 7 h to 48 h. After 48 h incubation, the TEER decreased to 62 ± 10% (negative control: 88 ± 4%). This effect on the barrier integrity was also confirmed by the significant (*p* < 0.001) increased permeability of ^14^C sucrose (Figure 4C). The permeability of the negative control with *p*_c_ (^14^C sucrose) = 0.42 ± 0.15 × 10^−6^ cm/s increased to 0.81 ± 0.17 × 10^−6^ cm/s after incubation with 1 µM OTA for 48 h (Figure 4C).

Furthermore, Figure 4A shows that 1 µM OTα (green line), CIT (yellow line), and DHCIT (orange line) have no significant effects on the TEER values. Incubation with 1 µM OTα resulted in TEER values of 92 ± 16% after 48 h. TEER values after 48 h incubation with 1 µM CIT and 1 µM DHCIT yielded 81 ± 10% and 79 ± 9% respectively. The absence of barrier-weakening effects of 1 µM OTα, CIT, and DHCIT on the BBB was confirmed by the permeability of ^14^C sucrose, illustrated in Figure 4C. None of the three mycotoxins caused a significant change. After 48 h incubation with 1 µM OTα the permeability was *p*_c_ (^14^C sucrose) = 0.56 ± 0.24 × 10^−6^ cm/s. Permeabilities after 48 h treatment with 1 µM CIT and DHCIT were *p*_c_ (^14^C sucrose) = 0.50 ± 0.33 × 10^−6^ cm/s and *p*_c_ (^14^C sucrose) = 0.38 ± 0.17 × 10^−6^ cm/s respectively (Figure 4C).

Besides the TEER values, the electrical capacitance *c*_CL_ of the cell monolayer was also monitored for 48 h (Figure 4B). As cellular bilayer membranes behave like an electrical capacitor, they influence the cellular impedance. Changes in *c*_CL_ are markers for the integrity of the cells [45]. Weak cytotoxic effects lead to a reduction of *c*_CL_, whereas strong cytotoxic effects result in an exponential increase in *c*_CL_ to more than 150% due to the usually observed detachment of the cells from the surface.

None of the four mycotoxins showed a significant change of *c*_CL_ compared to the negative control. After 48 h apical incubation with 1 µM OTA, OTα, CIT and DHCIT the *c*_CL_ reached 96 ± 4%, 98 ± 2%, 97 ± 4% and 99 ± 4% respectively, whereas the negative control (0.1% ACN) was maintained at 99 ± 2%. 

### 2.3. Transfer Studies

**Figure 5:**
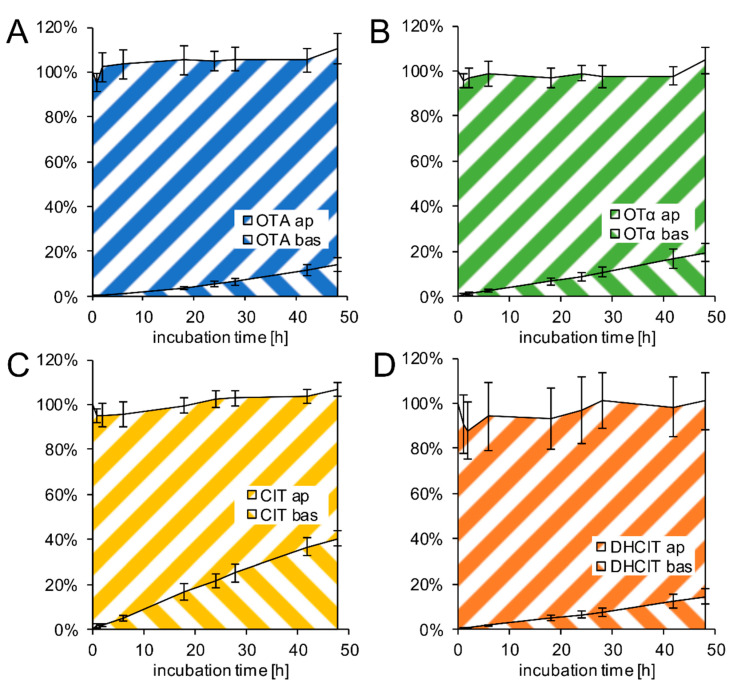
Transfer of 1 µM OTA (**A**), 1 µM OTα (**B**), 1 µM CIT (**C**), and 1 µM DHCIT (**D**) from the apical (ap) to the basolateral (bas) compartment after application on the apical side of PBCEC grown on Transwell^®^ filter for 48 h (*n* = 9). The concentrations are normalised to the initial amount of compound (760 pmol) in the apical compartment. The coloured lines from bottom left to top right represent amounts of the toxin in the apical compartment, whereas the coloured lines from top left to bottom right illustrate the toxin amounts in the basolateral compartment. The volumes of the apical and basolateral compartment are different and for this reason an equilibrium between ap and bas is reached at a ratio of 32%/68% (0.76 mL/1.65 mL).

For transfer studies from the apical (blood) side to the basolateral compartment (brain side), 1 µM of mycotoxins were applied on the apical side of the Transwell^®^ filter compartment. Over 48 h, OTA, OTα, CIT, and DHCIT were quantified at eight points in time in both compartments by HPLC-MS/MS in order to investigate the transfer and potential metabolism of the applied compounds. The results are shown in Figure 5. In addition, permeability coefficients were determined according to Equations (1) and (2) [9] and are given in Table 1.

All compounds show a continuous increase in the basolateral compartment and a simultaneous decrease in the apical compartment (Figure 5), indicating a transfer from the apical to the basolateral compartment. No metabolism was observed at the BBB, as the total recovery was around 100%. Furthermore, no conversion between OTA and OTα or between CIT and DHCIT was detectable by HPLC-MS/MS. Although the total recovery was almost 100%, the filter inserts including the cell monolayer were extracted and analysed by HPLC-MS/MS at the end of the experiment. The mycotoxin levels in the filter inserts were below 1%, indicating that the intracellular amounts are negligible.

As shown in Figure 5A, after 48 h incubation with 1 µM OTA 96 ± 7% of the apically applied OTA remained in the apical compartment and only 14 ± 3% were transferred to the basolateral compartment, leading to a permeability coefficient of *p*_c_ (OTA) = 0.38 ± 0.08 × 10^−6^ cm/s.

After the incubation with 1 µM OTα (Figure 5B) in the apical compartment, 86 ± 6% were quantified in the apical compartment after 48 h, and 19 ± 4% of the 760 pmol OTα were found to be transferred to the basolateral compartment. A permeability coefficient of *p*_c_ (OTα) = 0.68 ± 0.15 × 10^−6^ cm/s was calculated. 

Following the apical application of 1 µM CIT, 40 ± 4% were transferred over 48 h to the basolateral compartment (Figure 5C), whereas 67 ± 3% were recovered in the apical compartment. The permeability coefficient was *p*_c_ (CIT) = 1.79 ± 0.40 × 10^−6^ cm/s.

The incubation with 1 µM DHCIT (Figure 5D) gave similar results as observed for OTA. After 48 h, 87 ± 13% were detected in the apical compartment and 14 ± 3% on the basolateral side. Based on these results, a permeability coefficient of *p*_c_ (DHCIT) = 0.51 ± 0.14 × 10^−6^ cm/s could be calculated.

### 2.4. Active Transfer Studies

To analyse any active transport the mycotoxins were applied in the apical and basolateral compartments in equimolar concentrations of 200 nM. In a second set of experiments, 100 µM probenecid were added to both compartments 1 h before mycotoxin incubation in order to elucidate the effects of the OAT transport proteins. This concentration was previously shown to significantly inhibit multidrug resistance in leukaemia cell lines [46]. Higher levels of probenecid impaired the PBCEC barrier integrity (data not shown) and were not applied for this reason. The mycotoxin concentrations were analysed as described above by HPLC-MS/MS.

**Figure 6:**
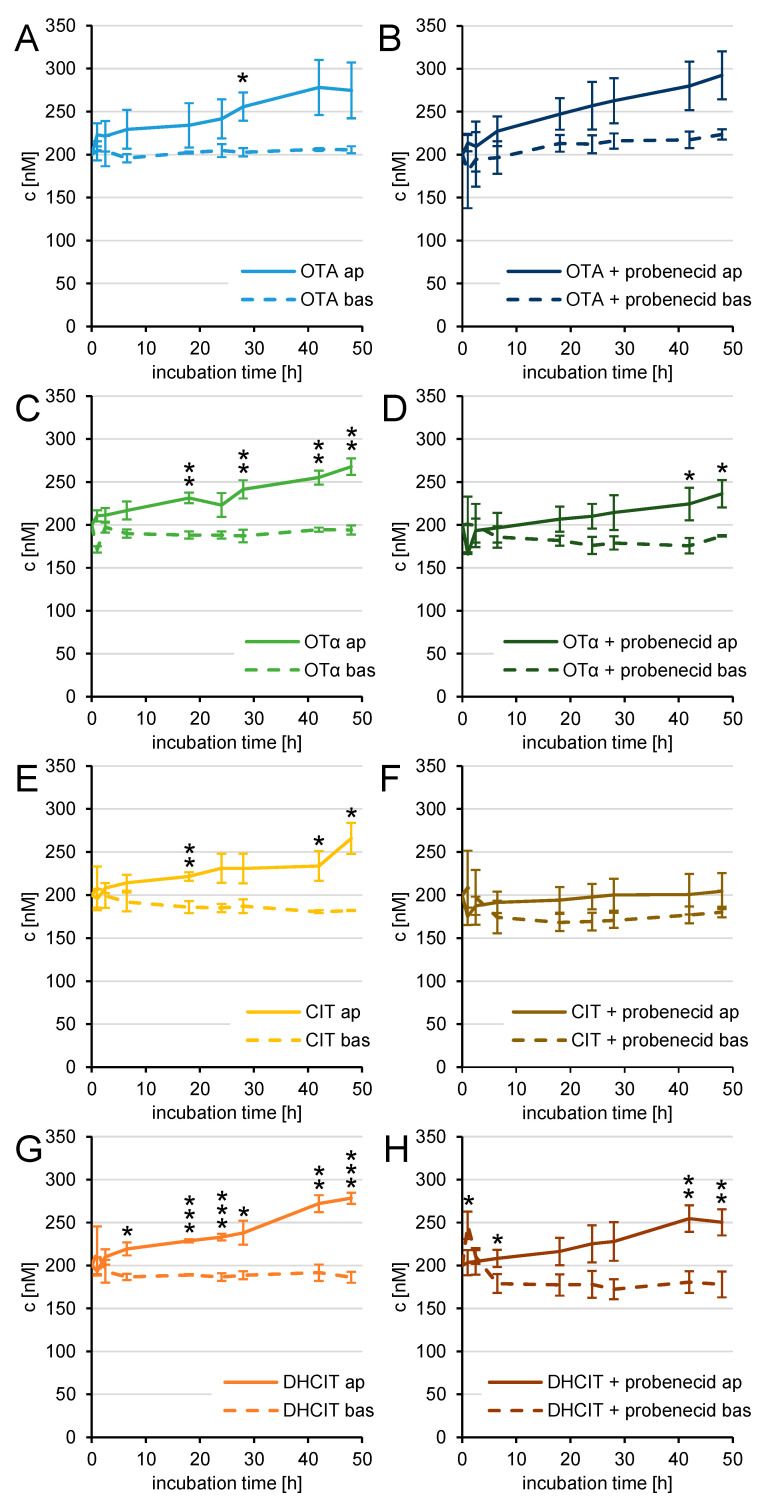
Recovery of 200 nM OTA (**A**,**B**), 200 nM OTα (**C**,**D**), 200 nM CIT (**E**,**F**), and 200 nM DHCIT (**G**,**H**) with or without the addition of 100 µM probenecid to the apical (ap) and basolateral (bas) compartment (*n* = 3) on a PBCEC monolayer. As the volumes of both compartments are different, the sum does not yield 400 nM. Significant differences between mycotoxin levels in both compartments are labelled (highly significant differences (*p* < 0.001) are marked with ***, differences with medium significance (*p* < 0.01) with ** and with low significance (*p* < 0.05) with *.

For OTA, shown in Figure 6A, an enrichment on the apical side was seen, which increases over time for most points in time. The difference between apical and basolateral compartment was significant (*p* < 0.05) at 28 h. After 48 h, 275 ± 32 nM OTA were recovered from the apical compartment, whereas 206 ± 4 nM OTA were detected on the basolateral side. As illustrated in Figure 6B, after a 1 h pre-incubation with 100 µM probenecid a similar relation was found, but the increase in the apical compartment reached no statistical significance at any given point in time. Forty-eight hours after application 292 ± 28 nM OTA was detected in the apical and 224 ± 6 nM OTA in the basolateral compartment. Therefore, probenecid had a minor almost negligible effect on the enrichment of OTA in the apical compartment.

Results of the incubation with OTα are displayed in Figure 6C. Forty-eight hours after the equimolar application of 200 nM OTα in both compartments 268 ± 10 nM OTα were quantified in the apical and 194 ± 5 nM OTα in the basolateral compartment. The increase in this enrichment was almost continuous. Significant differences (*p* < 0.01) between the two compartments were reached at 18 h, 28 h, 42 h, and 48 h. With 100 µM probenecid applied to both compartments beginning 1 h before the application of OTα, shown in Figure 6D, the enrichment was slightly weaker, reaching statistically higher levels (*p* < 0.05) in the apical compartment after 42 h and 48 h. At the end of the experiment after 48 h, 236 ± 16 nM OTα were found in the apical and 187 ± 1 nM OTα in the basolateral compartment. The effect of probenecid on the enrichment can therefore be considered as weak.

After equimolar incubation with 200 nM CIT in the apical and basolateral compartment, 266 ± 18 nM CIT were detected in the apical and 182 ± 1 nM CIT in the basolateral compartment (Figure 6E). An almost continuously increasing difference of CIT concentrations in both compartments was observed and was statistically significant after 18 h (*p* < 0.01), 42 h and 48 h (both *p* < 0.05). No significant differences between the CIT concentrations in both compartments were detected when 100 µM probenecid were incubated 1 h prior to CIT application as shown in Figure 6F. After 48 h, 205 ± 21 nM CIT were detectable in the apical and 180 ± 12 nM CIT in the basolateral compartment. Although the enrichment of CIT was not fully inhibited, some effect of probenecid cannot be dismissed.

Figure 6G displays the enrichment of DHCIT after 200 nM application in both compartments, which was significantly higher in the apical compartment after 6.5 h (*p* < 0.05), 18 h, and 24 h (both *p* < 0.001), 28 h (*p* < 0.05), 42 h (*p* < 0.01), and 48 h (*p* < 0.001). After 48 h, 278 ± 7 nM DHCIT were detected in the apical and 186 ± 6 nM DHCIT in the basolateral compartment. The enrichment of DHCIT after 200 nM equimolar incubation and pre-incubation with 100 µM probenecid in both compartments is shown in Figure 6H. The differences between the concentrations were slightly lower and significantly higher DHCIT concentrations in the apical compartment were detected after 6.5 h (*p* < 0.05), 42 h (*p* < 0.01), and 48 h (*p* < 0.01). After 1 h significantly (*p* < 0.05) more DHCIT was detected in the basolateral compartment, which is probably due to higher variations at the beginning of the experiment. Forty-eight hours after the application of 200 nM DHCIT, 250 ± 15 nM DHCIT were detected in the apical compartment, whereas 178 ± 15 nM DHCIT were quantified in the basolateral compartment.

As already observed after the transfer study described above, the mycotoxin amounts extracted from the cells and filter membranes were below 1% of the initially applied amount and can therefore be considered as negligible for the overall distribution of OTA, OTα, CIT and DHCIT (data not shown).

## 3. Discussion

### 3.1. Cellular Viability and Barrier Integrity

As mentioned in the previous section, OTα, CIT and DHCIT neither altered the cellular viability up to 10 µM nor impaired the barrier integrity nor the membrane integrity of PBCEC after incubation with up to 1 µM concentrations. In contrast, OTA showed stronger effects and reduced the cellular viability at 10 µM to less than 5% compared to the solvent control. In addition, 1 µM OTA caused a barrier impairment by a clear reduction of TEER values and a doubled permeation of ^14^C sucrose across the cell monolayer. Nevertheless, the PBCEC barrier was not completely disrupted and could therefore be used to analyse the OTA transfer across the PBCEC barrier.

Previous studies suggested neurotoxic effects of OTA in vivo [23] and in vitro [24,25,26,27,28,29]. Whereas the in vitro studies did not investigate the OTA uptake to the brain at all, it has to be considered that the administered OTA amounts in the in vivo study (289 µg/kg body weight/48 h to Wistar rats for up to 6 weeks [23]) were very high. Taking OTA bioavailability and exposure into consideration, high nanomolar or low micromolar blood levels might arise from this high intake, which is at least two orders of magnitude higher than the actual human exposure in the sub-nanomolar range [19,20]. In similar concentrations, barrier-weakening effects of OTA were observed in this study using PBCEC, as illustrated in Figure 4A,C. This barrier-weakening effect might have allowed the influx of potentially harmful compounds but also of OTA itself to the brain and explain the findings of the previously mentioned studies.

### 3.2. Transport Properties

In this study the transfer across a slightly or non-impaired PBCEC barrier was analysed, which was assured by continuous cellular impedance spectroscopy to exclude any diffusion of OTA through a leaky endothelial monolayer to the brain. Regarding their high lipophilicity, the very low transfer rates (Figure 5) and permeability coefficients (Table 1) of OTA, OTα and DHCIT are surprising. Only CIT showed a permeability, which exceeded the permeability of the negative control ^14^C sucrose by a factor of ca. 4. The permeability of CIT is therefore in a similar order of magnitude as morphine [47], which is known to cross the BBB to some extent. However, since morphine transfer is limited, CIT transfer to the brain is probably not very fast either.

In general, lipophilic molecules tend to permeate cellular barriers faster, because they usually have a higher membrane permeability. However, this does not seem to apply to the tested mycotoxins. A previous study showed a similar permeability for the more hydrophilic mycotoxins deoxynivalenol and moniliformin compared to ^14^C sucrose as the lipophilic CIT in this study [15]. This clearly shows that besides the lipophilicity other factors also influence the transfer across membranes and for this reason it is important to perform experimental transport studies. An estimation based on their lipophilicity alone would have predicted a much faster transfer rate and permeability of these mycotoxins compared to the polar compounds deoxynivalenol and moniliformin [15].

To investigate this result in more detail, the active transfer of OTA, OTα, CIT and DHCIT were studied as shown in Figure 6A,C,E,G. All four mycotoxins were enriched in the apical blood compartment, when equimolar concentrations (200 nM) were applied in both compartments. This indicates that the compounds are substrates of efflux transport proteins, which actively transport the mycotoxins from the brain back to the bloodstream to reduce the cerebral exposure to these compounds. These transport proteins are very likely causing the unexpectedly low transfer rates and permeability coefficients, observed in this study. In the light of these data, the proposed neurotoxic effects of the previously mentioned studies in vivo [23] and in vitro [24,25,26,27,28,29] seem to employ conditions that are prevented by the BBB and its transport barrier system in particular.

The specific update and enrichment of OTA in kidney cells of the proximal tube is an important factor of OTA induced nephrotoxicity and was subject to various studies [48,49,50]. Transport proteins of the solute carrier (SLC) family are the most important transporters responsible for the uptake of OTA. In particular, organic anion transporters (OAT) as OAT1 [51], OAT3 [52], OAT4 [53], and NTP4 [54], but also organic anion transporting polypeptides (OATP) such as OATP1A4 [55], as well as proton-dipeptide cotransporters [56], are involved in OTA enrichment in the kidney. Besides their existence in the kidney, several of these transport proteins are present at the apical or basolateral membrane of endothelial cells at the BBB, as well. Therefore, probenecid was used to inhibit these transporters and to investigate the effect of SLC class transporters on the efflux of the tested mycotoxins at the BBB. Probenecid was shown to inhibit the enrichment of OTA [57] and CIT [41] in the kidney and specifically inhibited the OTA transfer by OAT1 und OAT3 more effectively than other potential inhibitors [52].

By adding 100 µM probenecid to both compartments of the previously mentioned experimental setup to investigate the active transport, many transporters of the SLC class were inhibited. Although a slightly weaker enrichment of all mycotoxins was observed (Figure 6B,D,F,H), higher levels of OTA, OTα, CIT, and DHCIT were detected in the apical compartments even while co-incubating probenecid at almost every given point in time. These results suggest that transporters of the OAT class have only a minor impact on the efflux of the applied mycotoxins. This is in contrast to previous results obtained in kidney models, which identified these transporters as the most important for the transport of these mycotoxins. Only the enrichment of CIT was reduced slightly stronger by adding probenecid indicating a more important role of OAT in the efflux of CIT compared to OTA, OTα and DHCIT. Although no statistically significant differences were recorded, between 6.5 h and 48 h CIT levels in the apical compartment were continuously higher compared to the CIT levels in the basolateral compartment while co-incubating 100 µM probenecid.

Studies using Caco-2 cells to investigate the transport properties at the intestinal barrier revealed multidrug resistance-associated protein 2 (MRP2) and BCRP as the most important efflux transporters [58]. Since both transporters are frequently found at the BBB, they might contribute to the efflux of the tested mycotoxins in the PBCEC model. However, previous studies showed that probenecid inhibits some MRP transporters and especially MRP1, MRP2, and MRP4, which are also present at the BBB [59,60]. Therefore, it seems more likely that BCRP is the major efflux transporter for OTA, OTα, CIT, and DHCIT at the BBB. Further studies might address this question to clarify which transport protein is mainly responsible for the efflux of these mycotoxins at the BBB. However, it is also likely that not just one but several transporters might be involved in OTA, OTα, CIT, and DHCIT efflux.

Besides the low transfer rates found in this study, the strong serum-binding properties of OTA [61,62] and CIT [63] have to be considered, if comparing the results to the in vivo situation. Whereas this model using PBCEC relies on serum-free conditions, to keep the TEER values as high and the permeability of the cell monolayer as low as possible [10], the human blood obviously contains high amounts of human serum albumin (HSA). Compounds with strong serum-binding properties penetrate the BBB slower than those with weak or no serum-binding properties, since unbound compounds cross cellular membranes much easier [1]. The transfer of all four analysed toxins might therefore be even slower and the corresponding cerebral exposure to OTA, OTα, CIT and DHCIT in vivo even lower.

## 4. Conclusions

In this study, data on the effects of OTA, OTα, CIT and DHCIT on the BBB are presented. The used PBCEC model is a well-established system to study effects on the BBB in vitro [8,9]. Although the obtained results may not be directly transferred to the in vivo situation, they give hints on the potential neurotoxic effects of mycotoxins.

In this study, 10 µM OTA exhibited cytotoxic and 1 µM OTA barrier-weakening effects on PBCEC, whereas OTα, CIT and DHCIT did not impair barrier integrity or cause cytotoxic effects.

All four mycotoxins crossed the BBB model in vitro, but the amounts of OTA, OTα, and DHCIT, which were transferred to the basolateral (brain) compartment, were very low and within the same order of magnitude as the negative permeability marker ^14^C sucrose. Sucrose is known not to cross the BBB in vivo. Therefore, it is unlikely for these compounds to cross the BBB in vivo as well. However, CIT transfer was slightly faster and exceeded the permeability of ^14^C sucrose by more than a factor of four. 

The binding of xenobiotics to HSA in the blood generally reduces their blood-to-brain transfer in vivo [1]. Since OTA [61,62] and CIT [63] strongly bind to HSA, it is likely that their transfer to brain in vivo is even lower than the results presented in this in vitro study. Investigating the influence of HSA on mycotoxin transfer to the brain might be an interesting subject for further studies, but PBCEC are very sensitive to high serum levels, which drastically reduce TEER values and increase permeability across the cell monolayer [10]. Therefore, other models might be more suitable to investigate the influence of HSA on OTA and CIT transfer at the BBB.

Nevertheless, the transfer rates and permeabilities of all four mycotoxins were much lower than one might assume regarding their molecular structure and high lipophilicity. The reason for this surprising result can be attributed to efflux transport proteins, which transport their substrates back to the blood stream, or the apical compartment in the presented model.

Probenecid was used to inhibit OAT, which contribute most to the excretion of OTA and CIT in renal tissues. Unexpectedly, the co-incubation with probenecid had no or only minor effects, on the enrichment of OTA, OTα, CIT and DHCIT in the apical compartment. This leads to the hypothesis, that OAT play a subordinate role in the efflux of these four molecules. Earlier studies in Caco-2 cells found BCRP and MRP2 to be involved in the efflux of OTA at the intestinal barrier in vitro [58]. Since both proteins are also responsible for the efflux at the BBB, it is likely that especially BCRP is at least partly responsible for the low cerebral exposure of the brain towards OTA, OTα, CIT, and DHCIT, as well.

The presented data lead to the conclusion, that none of the four analysed mycotoxins are of particular concern for human cerebral health, assuming a regular diet and normal exposure to OTA, OTα, CIT, and DHCIT. OTA and CIT might only be of concern in case of a defective BBB, which increases the transfer of the mycotoxins to the brain. Besides genetic predisposition, this barrier impairment can also be caused by other mycotoxins such as DON, as shown in a previous study [15]. Another situation increasing cerebral exposure to these mycotoxins would be high blood levels of transport protein inhibitors, as they are present during chemotherapy to overcome the multiple drug resistance of cancer cells.

## 5. Materials and Methods

### 5.1. Chemicals and Reagents

All chemicals for mycotoxin quantification were obtained from VWR International GmbH (Darmstadt, Germany), Grüssing GmbH Analytica (Filsum, Germany) and Sigma-Aldrich Chemie GmbH (Steinheim, Germany). Cell culture media and supplements were purchased at Biochrom AG (Berlin, Germany) and PAA Laboratories GmbH (Pasching, Austria). Water was from a Milli-Q Gradient A10 system (Merck KGaA, Darmstadt, Germany).

OTA with a purity of ≥98% was isolated and hydrolysed to OTα as previously described [64]. CIT with a purity of ≥97% (HPLC) was purchased at Apidogen AG (Liestal, Switzerland). DHCIT was purchased at AnalytiCon Discovery GmbH (Potsdam, Germany). T-2 toxin with >90% purity (^1^H NMR) was isolated as described [65]. For the following experiments, 1 mM stock solutions of each mycotoxin were prepared in acetonitrile (ACN) and stored at −20 °C.

### 5.2. Viability Test and PBCEC Cell Culture

To ensure that the applied mycotoxin concentrations show no cytotoxic effects, PBCEC were incubated with 10 nM to 10 µM of each toxin, as previously described [15]. Cellular viability was tested after 48 h using the cell counting kit 8 (CCK-8, Donjindo Laboratories, Tokyo, Japan). The detailed protocol for viability testing is based on our previous publication [15] and can be found in the Supplementary Materials. The general procedure for the cultivation and handling of PBCEC is described in detail in literature [9].

### 5.3. Transfer Studies

The transfer studies using the PBCEC model were performed according to our previous study [15]; the sole exception was the tenfold reduction of the mycotoxin concentration applied to the apical compartment to 1 µM. The detailed protocol can be found in the Supplementary Materials.

### 5.4. Active Transport Studies

For active transport studies, the cells were treated as described for transfer studies, with the only difference being that the compounds were applied on the apical and basolateral side in equimolar 200 nM concentrations (see Supplementary Materials). To investigate whether probenecid has an effect on the active transfer of the mycotoxins via OAT, 100 µM probenecid was applied to the apical and basolateral compartment 1 h before mycotoxin application and maintained until the end of the experiment. The changes in concentration were monitored 1, 2.5, 6.5, 18, 24, 28, 42, and 48 h after applying the mycotoxins using HPLC-MS/MS.

### 5.5. Barrier Integrity

For barrier integrity, TEER values were monitored using a cellZscope^®^ cellular impedance spectrometer (nanoAnalytics, Münster, Germany). Furthermore, the permeability of ^14^C sucrose was measured. The detailed protocols can be found in the Supplementary Materials.

### 5.6. Mycotoxin Quantification

The quantification of OTA, OTα, CIT, and DHCIT by HPLC-MS/MS was performed with a 1290 Infinity series (Agilent Technologies, Santa Clara, CA, USA) liquid chromatograph coupled to a QTRAP 6500 (SCIEX Germany GmbH, Darmstadt, Germany) mass spectrometer. Both devices were operated with Analyst 1.6.2 software (SCIEX Germany GmbH, Darmstadt, Germany). Chromatographic separation of OTA, OTα, CIT, and DHCIT was performed on a 100 mm × 2.1 mm Halo RP-Amide 2.7 µm column (Advanced Materials Technology, Wilmington, NC, USA) equipped with an Ultra filter (KrudKatcher, Phenomenex, Aschaffenburg, Germany) at 40 °C using a gradient of ACN+1% formic acid (FA) (Solvent A) and H_2_O+1% FA (solvent B) at a flow rate of 250 µL/min. The gradient started with 10% A, held for 4 min, raised to 50% A at 5 min, further raised to 61% at 10 min, and raised to 100% and held until 11 min. Afterwards, the column was re-equilibrated for 4 min. An injection volume of 5 µL was used. A diverter valve was used to exclude the first 5 min of the HPLC run from the mass spectrometer and reduce matrix contamination. The Ion Drive Turbo V ion source was heated to 400 °C. Further conditions were curtain gas (CUR) 35 psi N_2_, nebuliser gas (GS1) 45 psi N_2_, heater gas (GS2) 55 psi N_2,_ and ion spray voltage 4500 V in positive and −4500 V in negative ionization mode; each MRM transition was monitored for 5 ms. Detailed mass spectrometer settings are given in the Supplementary Materials of this article.

To quantify the mycotoxin levels during the transfer studies, eight calibration solutions ranging from 5 nM to 1 µM diluted in preincubated serum-free PBCEC medium (550 nM hydrocortisone) were analysed at least three times per analysis of one cell preparation. For the active transport studies, five standards ranging from 100 nM to 300 nM were used. Data processing was performed with Analyst 1.6.2 (SCIEX Germany GmbH, Darmstadt, Germany) using linear regression with R^2^ < 0.99.

### 5.7. Permeability Calculations

The permeability coefficients *p* were calculated according to (1) as described in detail in [15]:

(1) $$p\left[\mathrm{cm}/s\right]=\frac{c_{\mathrm{bas}}\left[\%\right]}{c_{0\;h,\;\mathrm{bas}}\left[\%\right]}\cdot \frac{V_{\mathrm{ap}}\left[\mathrm{cm}³\right]}{A\left[{\mathrm{cm}}^{2}\right]\cdot t\left[s\right]}$$

where *c*_bas_ is the mycotoxin concentration in the basolateral compartment at a certain time *t* [s]. The initial concentration in the apical compartment is *c*_0 h,ap_ and *V*_ap_ is the volume of the compartment at the beginning of the experiment. *A* is the area of the filter insert membrane. The resulting permeability of the mycotoxin across the cell monolayer *p*_c_ was calculated as shown in (2):

(2) $$\frac{1}{p_{c\;}\left[\mathrm{cm}/s\right]}=\frac{1}{p_{c+f\;}\left[\mathrm{cm}/s\right]}\cdot \frac{1}{p_{f}\left[\mathrm{cm}/s\right]}$$

where *p*_c+f_ is the permeability coefficient of the transfer study and *p*_f_ the permeability coefficient of the cell-free polycarbonate filter membrane [9].

### 5.8. Statistics

All data were statistically evaluated by Excel 2013 (Microsoft Corporation, Redmond, WA, USA). Unpaired heteroscedastic Student’s T-test was used to calculate significant differences between data sets. Highly significant differences (*p* < 0.001) are marked with ***, medium significance (*p* < 0.01) with **, and with low significance (*p* < 0.05) with *.

The viability test was conducted in three different preparations, each consisting of six individual replicates (*n* = 18). Transport studies including TEER and *c*_CL_ analysis were performed in three replicates in three independent preparations (*n* = 9). Studies aiming for the active transfer and effects of probenecid used three preparations with one replicate each (*n* = 3). ^14^C sucrose permeability was analysed six times per filter in three replicates in one preparation (*n* = 18).

## Figures and Tables

**Table 1:** Permeability coefficients of OTA, OTα, CIT, and DHCIT and ^14^C sucrose (negative permeability marker) from the apical to the basolateral side of a PBCEC monolayer (*n* = 9).

Compound	*p*_c_ [10^−6^ cm/s]	*t* [h]
^14^C sucrose	0.43 ± 0.20	0.17–1.33
1 µM OTA	0.38 ± 0.08	18
1 µM OTα	0.68 ± 0.15	18
1 µM CIT	1.79 ± 0.40	18
1 µM DHCIT	0.51 ± 0.14	18

## Supplementary Materials

The following are available online at https://www.mdpi.com/article/10.3390/toxins13050327/s1, Figure S1: Lightmicroscopic images of PBCEC after 48 h incubation of OTA, OTα, CIT, DHCIT, negative and positive controls. Furthermore, methodical details on the LC-MS/MS settings used for mycotoxin quantification and detailed cell culture protocols are provided.
