# Supplementary material for: Efflux at the Blood-Brain Barrier Reduces the Cerebral Exposure to Ochratoxin A, Ochratoxin α, Citrinin and Dihydrocitrinone
Source: Toxins (Basel). 2021 Apr 30;13(5):327. doi: 10.3390/toxins13050327 (PMC8147254; doi:10.3390/toxins13050327)
Supplement: Supplementary file 1 [file toxins-13-00327-s001.zip › toxins-1195930-supplementary.pdf]

Supplementary Materials: Efflux at the Blood-Brain Barrier Reduces the Cerebral Exposure to Ochratoxin A, Ochratoxin α, Citrinin and Dihydrocitrinone

Matthias Behrens, Sabine Hüwel, Hans-Joachim Galla and Hans-Ulrich Humpf

1. Microscopic Images

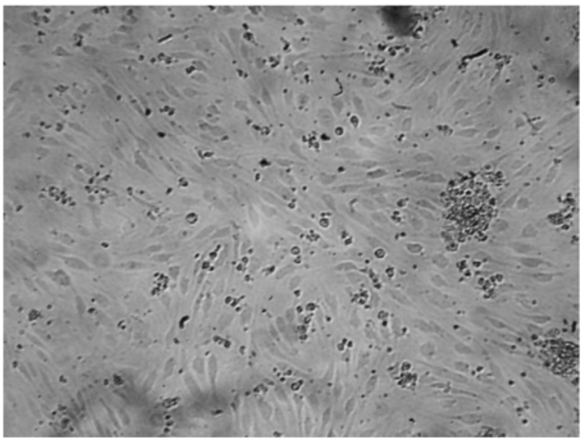

A

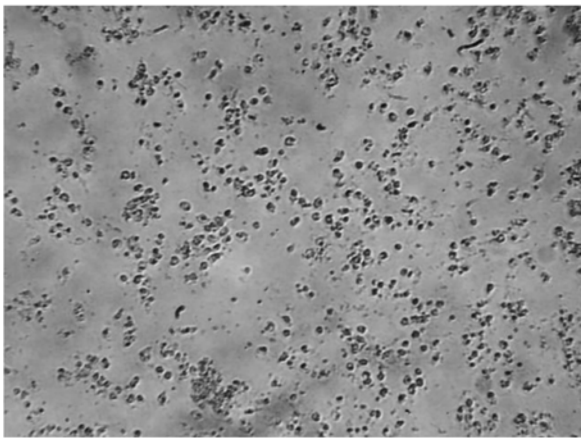

B

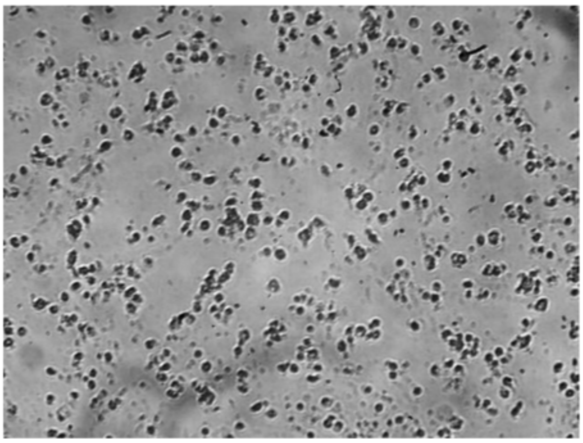

C

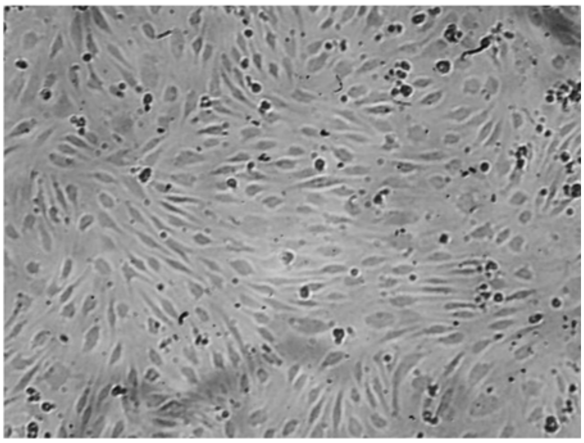

D

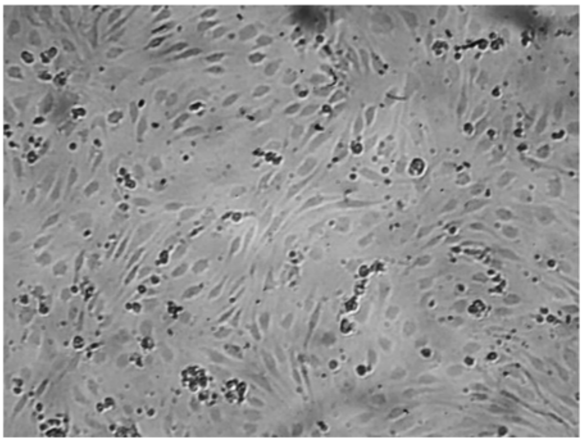

E

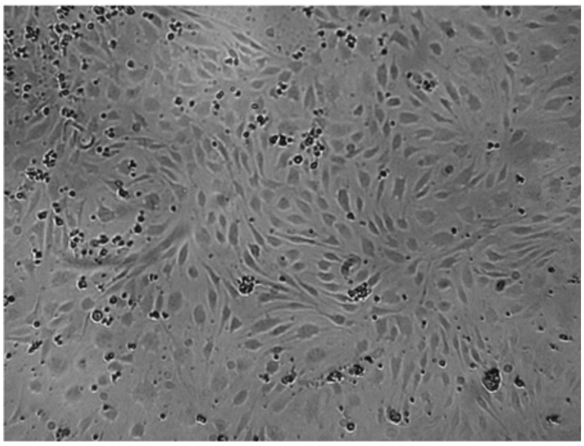

F

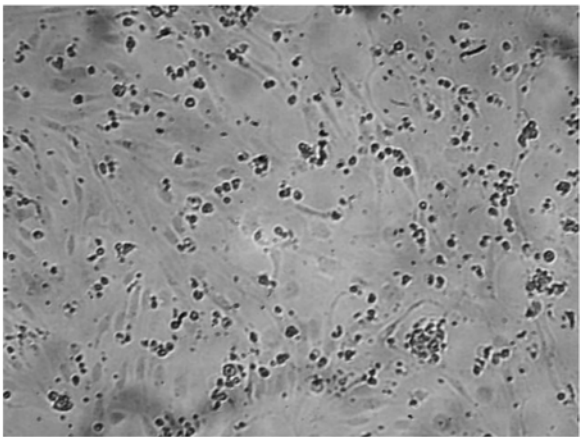

G

Figure S1. Lightmicroscopic images of PBCEC after 48 h incubation with OTA, OT*α*, CIT, DHCIT, negative and positive controls. Compared to the negative control (0.1% ACN), incubations with 10 µM OTA and 10 µM T 2 toxin (positive control) clearly showed much smaller spherical cells, which are partly detached from the cell culture vessel and no typical endothelial spindle-shaped cell monolayers. However, incubations with 10 µM OT*α*, CIT and DHCIT yielded unremarkable morphologies compared to the negative control. 1 µM OTA caused more small spherical cells, but an endothelial cell monolayer was present. Images were taken at 40× magnification. All images are shown in greyscale with contrast increased by 40% and were obtained by MEM1300 camera (Future Optics Sci. & Tech. Co. Ltd., Hangzhou, China) with Future WinJoe 1.0.7.91 software. (**A**) Negative control (0.1% ACN) after 48 h; (**B**) Positive control (10 µM T-2 toxin) after 48 h; (**C**) 10 µM OTA after 48 h; (**D**) 10 µM OT*α* after 48 h; (**E**) 10 µM CIT after 48 h; (**F**) 10 µM DHCIT after 48 h; (**G**) 1 µM OTA after 48 h.

2. Detailed LC-MS/MS settings

OTA was analysed in the positive ionization mode with a declustering potential (DP) of 35 V, an entrance potential (EP) of 10 V and a collision cell exit potential (CXP) of 11 V. The MRM transition *m*/*z* 404.1→*m*/*z* 239.0 with a collision energy (CE) of 31 V was used as quantifier, whereas the OTA structure was confirmed by the MRM transition *m*/*z* 404.1→*m*/*z* 221.0 with a CE of 48 V. For the quantification of OT*α* in the negative ionization mode the MRM transition *m*/*z* 255.0→*m*/*z* 167.0 was used with a DP of −31 V, EP −6 V, CE −33 V and CXP −8 V. Identity of OT*α* was confirmed using the MRM transition *m*/*z* 257.0→*m*/*z* 221.0 in the positive ionization mode with a DP of 29 V, EP 10 V, CE 30 V and CXP 12 V. CIT was analysed in the positive ionization mode with a DP of 61 V and EP 10 V. Quantification was performed using the MRM transition *m*/*z* 251.0→*m*/*z* 205.0 with a CE of 37 V and CXP 12 V. For qualitative information MRM *m*/*z* 251.0→*m*/*z* 115.0 was analysed with a CE of 67 V and CXP 14 V. DHCIT analysis was carried out in both polarities. To quantify DHCIT in the positive ionization mode the MRM transition *m*/*z* 267.0→*m*/*z* 231.1 with a DP of 54 V, EP 5 V, CE 34 V and CXP 13 V was used. To confirm the molecular structure of DHCIT the MRM transition *m*/*z* 265.0→*m*/*z* 221.1 with DP −15 V, EP −10 V, CE −26 V and CXP −12 V were used.

3. Viability test and PBCEC cell culture

Cellular viability was tested after 48 h using the cell counting kit 8 (CCK-8, Donjindo Laboratories, Tokyo, Japan). The general procedure for cultivation of PBCEC is based on an earlier protocol [1]. After gentle resuspension in complete medium (Medium 199 Earle's with 100 U/mL penicillin, 100 µg/mL streptomycin, 100 µg/mL gentamycin, 4.1 mM L-glutamine and 10% fetal calf

serum) and centrifugation at 220 × *g* at 20 °C for 10 min the supernatant was removed. Fresh complete medium was added and the cell pellet was gently resuspended twice to a final concentration of 150,000 cells/mL. 100 µL of this suspension were seeded on rat-tail collagen coated 96-well plates (rat-tail collagen was isolated as described previously [1]) resulting in 15,000 cells per well. PBCEC were incubated at 37 °C, 5% CO_2 in a saturated humidified atmosphere. After 48 h the medium was replaced with serum-free medium (DMEM/Ham's F-12 with 100 U/mL penicillin, 100 µg/mL streptomycin, 100 µg/mL gentamycin, 0.7 mM L-glutamine and 550 nM hydrocortisone (Sigma-Aldrich, Steinheim, Germany)). 96 h after seeding 50 µL of the medium were removed and replaced with 50 µL double concentrated mycotoxin solution to achieve final concentrations of 10 nM to 10 µM with a maximum ACN concentration of 1%. After 48 h incubation with the toxin, 10 µL CCK-8 solution were added to each well and incubated for 70 min at 37 °C, 5% CO_2 in a saturated humidified atmosphere according to the manufacturers manual. After the incubation, the absorbance of the formed formazan dye was analyzed at 457 nm with 650 nm as a reference using an Infinite M200 PRO microplate reader with Tecan i-control software version 1.7.1.12 (Tecan, Crailsheim, Germany). The reference absorption was subtracted from the formazan absorption. Afterwards a blank absorption without cells was subtracted. Viabilities are normalized to a solvent adjusted negative control, which was not incubated with any mycotoxin.

4. Transfer studies

The thawing and seeding procedure was performed according to the protocol already described for the viability test as previously published [2]. Instead of using 96-well plates, 500 µL PBCEC suspension (500,000 cells/mL) were seeded in the upper (apical) compartment of a 12-well Transwell® polycarbonate filter membrane (Corning, Wiesbaden, Germany) with a growth area of 1.13 cm^2 (250,000 cells/filter). The lower (basolateral) compartment was filled with 1.5 mL complete medium. Medium was exchanged with serum-free medium after 48 h, as described before. 96 h after seeding the volume of the apical compartment was filled up to 760 µL. The Transwell® filters were briefly transferred to the cellZscope® cellular impedance spectroscope (nanoAnalytics, Münster, Germany) equipped with a 24 12-well Transwell® filter setup. Each well was previously filled with 1650 µL serum-free medium. Transendothelial electrical resistances (TEER) were analyzed using the cellZscope® device. Only Transwell® filters with TEER values of more than 600 $\Omega \cdot cm^2$ and electrical capacitances (c_{CL}) of 0.4 to 0.6 $\mu F/cm^2$ were used for transfer studies. 76 µL of the medium in the apical compartment were exchanged with 76 µL of tenfold concentrated mycotoxin solution. The final concentration of the mycotoxins was 1 µM at the beginning of the mycotoxin incubation. The final solvent concentration was 0.1% ACN. Incubation began with 760 µL medium in the apical and 1650 µL medium in the basolateral compartment as recommended by the cellZscope® manufacturer. To quantify the amount of the transferred mycotoxins, samples were collected 1, 2.5, 6.5, 18, 24, 28, 42 and 48 h after toxin application. To maintain the 1:2.17 ratio of the compartments, the apical samples were 46 µL whereas 100 µL samples were collected from the basolateral compartment. After 48 h the experiment was stopped, and the polycarbonate filter membranes were cut out of the Transwell® inserts with a scalpel, washed with PBS (without Ca^{2+} and Mg^{2+}), and extracted with ACN/H_2O (4+1, v/v) in an ultrasonic bath for 1 h. The extract was dried under nitrogen atmosphere and reconstituted in ACN/H_2O (1+9, v/v). The collected samples and filter extracts were analyzed and quantified using high performance liquid chromatography-tandem mass spectrometry (HPLC-MS/MS) or high performance liquid chromatography-high resolution mass spectrometry (HPLC-HRMS) respectively. Transfer rates were calculated as a fraction of the measured amount in the basolateral compartment and the total amount, which was incubated at the beginning of the experiment in the apical compartment.

5. Active transport studies

To study the active transfer properties, the cells were treated in the same manner as for the normal transfer studies, but the mycotoxins were incubated on the apical and basolateral side in equimolar 200 nM concentrations as previously described [2]. To investigate whether probenecid has an effect on the active transfer of the mycotoxins via OAT, 100 µM probenecid was applied to the apical and basolateral compartment 1 h before mycotoxin application and maintained until the end of the experiment. The changes in concentration were monitored 1, 2.5, 6.5, 18, 24, 28, 42 and 48 h after applying the mycotoxins using HPLC-MS/MS.

6. Barrier integrity

6.1. Cellular impedance spectroscopy

To monitor the barrier integrity during all transfer studies, the transendothelial electrical resistance (TEER) was measured with a cellZscope® cellular impedance spectrometer (nanoAnalytics, Münster, Germany) using a module suitable for 24 Transwell® filter inserts. After the mycotoxins were added to the cell culture medium, TEER was analyzed until the end of the experiment (48 h). The waiting time after each run was set to 10 min, resulting in 60 scans per 48 h. The results were normalized to the TEER values of the first impedance spectrum of each well. Data processing was performed using the cellZscope 1.3.4 software (nanoAnalytics, Münster, Germany).

6.2. ^{14}C sucrose permeability

As additional method to control the barrier tightness the permeability of radiolabeled ^{14}C sucrose across the Transwell® inserts was measured. Since ^{14}C sucrose is not transported or taken up by endothelial cells, the permeability of ^{14}C sucrose is an appropriate parameter for barrier integrity. After 48 h exposure to the mycotoxins 1 µCi (37 kBq) ^{14}C sucrose (Amersham, Buckinghamshire, UK) was applied to the apical compartment. Samples from the basolateral compartment were collected after 10, 20, 30, 40, 60 and 80 min, diluted in scintillation fluid and analyzed with a scintillation counter as previously described [1].

References

1. Franke, H.; Galla, H. J.; Beuckmann, C. T. An improved low-permeability in vitro-model of the blood-brain barrier: transport studies on retinoids, sucrose, haloperidol, caffeine and mannitol. *Brain Res.* **1999,** *818* (1), 65–71. DOI: 10.1016/S0006-8993(98)01282-7.
2. Behrens, M.; Hüwel, S.; Galla, H.J.; Humpf, H.U. Blood-Brain Barrier Effects of the Fusarium Mycotoxins Deoxynivalenol, 3 Acetyldeoxynivalenol, and Moniliformin and Their Transfer to the Brain. *PLoS ONE* **2015,** *10* (11), 1–20. DOI: 10.1371/journal.pone.0143640.
